# Recent Advances in Remote Pulmonary Artery Pressure Monitoring for Patients with Chronic Heart Failure: Current Evidence and Future Perspectives

**DOI:** 10.3390/s23031364

**Published:** 2023-01-26

**Authors:** Pascal R. D. Clephas, Dilan Aydin, Sumant P. Radhoe, Jasper J. Brugts

**Affiliations:** Department of Cardiology, Thorax Center, Erasmus University Medical Center, 3015GD Rotterdam, The Netherlands

**Keywords:** CardioMEMS, Cordella, telemonitoring, e-health, remote care, heart failure, sensor, hemodynamics

## Abstract

Chronic heart failure (HF) is associated with high hospital admission rates and has an enormous burden on hospital resources worldwide. Ideally, detection of worsening HF in an early phase would allow physicians to intervene timely and proactively in order to prevent HF-related hospitalizations, a concept better known as remote hemodynamic monitoring. After years of research, remote monitoring of pulmonary artery pressures (PAP) has emerged as the most successful technique for ambulatory hemodynamic monitoring in HF patients to date. Currently, the CardioMEMS and Cordella HF systems have been tested for pulmonary artery pressure monitoring and the body of evidence has been growing rapidly over the past years. However, several ongoing studies are aiming to fill the gap in evidence that is still very clinically relevant, especially for the European setting. In this comprehensive review, we provide an overview of all available evidence for PAP monitoring as well as a detailed discussion of currently ongoing studies and future perspectives for this promising technique that is likely to impact HF care worldwide.

## 1. Introduction

With 26 million patients worldwide almost a decade ago, heart failure (HF) was then already described as a global pandemic [1]. This global pandemic has continued to grow over the past years, resulting in an astounding 64.3 million HF patients worldwide based on more recent data [2,3]. Considerable progress has been made in HF therapy, which includes several guideline recommendations for HF treatment that have been proven to be life-saving in large randomized controlled trials (RCTs) [4,5]. However, despite improvement of survival rates of HF, the mortality remains high with estimated survival rates of 56.7% and 34.9% for 5 and 10 years, respectively [6]. In addition, HF is also a major cause of recurrent hospitalizations [1], which are associated with poor prognosis [7,8]. The healthcare costs associated with HF are substantial ($24,383 per patient annually in the US) and are mainly driven by the high costs of HF-related hospitalizations ($15,879) [9]. HF thus represents a major healthcare problem both from a clinical and economic perspective.

Remote monitoring of congestion markers has been proposed as a way to detect worsening HF patients in an earlier stage, which makes timely pharmacological intervention possible to avoid hospitalization [10]. Most decompensated HF hospitalizations and rehospitalizations are indeed presented with overt clinical congestive symptoms [11], which emphasizes the need to monitor congestion markers such as volume changes, preferably outside the hospital and in a preventive setting. Since non-invasive markers such as signs, symptoms, and weight change are a late sign of clinical congestion and have been reported to be poorly correlated with filling pressures [12], invasive methods should be better suited for this purpose as they can provide hemodynamic data which functions as a better marker for filling pressures [13,14]. Filling pressures are also the central target of many HF medications, especially diuretics.

Pulmonary artery pressure (PAP) monitoring is a form of hemodynamic-guided HF management and involves an implantable sensor which can measure PAP remotely [10,15]. Congestion is associated with an increase in PAP, often even before clinical symptoms, and elevated PAP is associated with a higher risk of HF events, typically seen days or weeks prior to the occurrence of a HF event [16,17]. PAP is therefore a promising early congestion marker to detect impeding HF-related hospitalizations. Additionally, a decrease in PAP is associated with a reduction of HF-related hospitalizations, irrespective of left ventricular ejection fraction (LVEF) [18,19], with a proportionally larger decrease in PAP being associated with a greater reduction of HF hospitalizations [20]. Currently, two types of PAP monitoring devices are used for PAP monitoring, namely the CardioMEMS HF system (Abbott, Sylmar, CA, USA) and Cordella PAP Sensor (Endotronix, Inc., Chicago, IL, USA). The CardioMEMS HF system is FDA approved and CE marked. The Cordella HF system is also FDA approved, and results of the CE mark trial have recently been published [21].

New evidence for PA pressure monitoring has emerged and to date, not only the CardioMEMS HF System is available for PAP monitoring, but also the Cordella System. Still, randomized clinical trial data are currently only available for the CardioMEMS HF system. Furthermore, there have been significant changes in HF therapy with the addition of new drug classes in the European HF guidelines [5]. In this comprehensive review, we provide an overview of all available evidence for both systems as well as a detailed discussion of currently ongoing studies and future perspectives for this promising technique that is likely to impact HF care worldwide.

## 2. Methods

The Medline database was searched for studies mentioning ‘CardioMEMS’ and ‘Cordella’ in title or abstract up until December 2022. Clinical studies assessing either or both of these devices in HF patients were selected, while case reports and case series were excluded. The references of relevant literature studies as well as the selected clinical studies were searched for additional studies. The results of the included studies were qualitatively summarized in a CardioMEMS HF system section and a Cordella PAP sensor section. Finally, a section on the role of PAP monitoring in assessing hemodynamic effects of medical therapy was included. Studies are clustered in paragraphs based on design.

## 3. Current Evidence

### 3.1. CardioMEMS HF System

#### 3.1.1. Device Information

**Figure 1:**
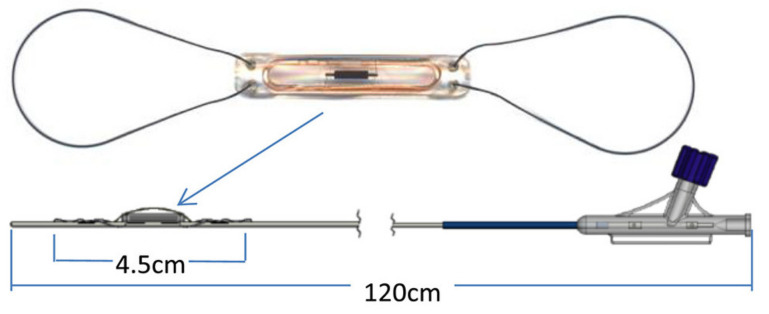
The CardioMEMS HF system pulmonary artery pressure sensor. Reprinted from ESC Heart Failure Volume 7, Issue 3, Cowie et al., Rationale and design of the CardioMEMS Post-Market Multinational Clinical Study: COAST, pages 865–872, Copyright 2020, with permission from Wiley [23].

The details of the CardioMEMS HF system technology have been elaborately described elsewhere [22]. In brief, the CardioMEMS HF system is an implanted sensor designed for placement in the (left) pulmonary artery (Figure 1). The sensor works without batteries since it uses radio frequent energy from the measurement device that is used to take daily readings [22]. It has no interference with implanted cardiac resynchronization therapy (CRT) or defibrillator devices (ICD) [22]. Patients are instructed to take daily readings with the device which has the form of a pillow and connects to the sensor in the body. A single reading takes approximately half a minute. It records mean PAP (mPAP), systolic PAP (sPAP), and diastolic PAP (dPAP), and pulse. The physician can make treatment decisions based upon trends in the mPAP or dPAP and can set specific targets and thresholds for alarms and notifications.

#### 3.1.2. Randomized Controlled Trials

The first RCT assessing the safety and efficacy of CardioMEMS HF system was the CHAMPION trial, performed more than a decade ago [24]. CHAMPION was at the time the first revolutionary RCT assessing such a technology with clinically meaningful endpoints and was also praised for the overall design of the trial [25]. Patients with chronic HF, New York Heart Association (NYHA) class III, and a HF-hospitalization in the previous year were eligible for inclusion, irrespective of left ventricular ejection fraction (LVEF). The efficacy for the CardioMEMS HF system in reducing HF hospitalizations was shown with 84 HF-related hospitalizations in the treatment group (n = 270) versus 120 in the control group (n = 280) (hazard ratio (HR) 0.72; 95% CI 0.60–0.85; *p* = 0.0002) in the first six months of follow-up and 158 versus 254 (HR 0.63; 95% CI 0.52–0.77; *p* < 0.0001) in a mean follow-up of 15 months [24]. A shortcoming of this study was the absence of information on drug changes, which includes drug-related adverse events considering the risk of overly aggressive treatment with diuretics and vasodilators [25]. Five years later, the complete follow-up results of CHAMPION were published where they extended the randomized access period with a mean follow-up of 18 months to an open access period with a mean follow-up of 13 months [26]. The randomized access period resulted in a consistent decrease of HF-hospitalizations of 33% in the treatment group compared with the control group (HR 0.67; 95% CI 0.55–0.80; *p* < 0.0001) [26]. The open access period, where the control group essentially crossed-over to the treatment group, resulted in a reduction in HF-hospitalizations of 48% as compared to the randomized access period (HR 0.52; 95% CI 0.40–0.69; *p* < 0.0001) [26]. In addition, they reported the previously missing pharmacological changes in diuretics, neurohormonal antagonists, and vasodilators for both groups. The number of changes for each drug class was evidently greater in the treatment group [26], which is likely what mediated the improved outcomes considering timely pharmacological intervention is what PAP monitoring was meant for. Especially this finding is what made the benefit of remote PAP monitoring convincing, with the side note that coordinated efforts are needed to properly integrate this in clinical practice and routine care [27].

**Figure 2:**
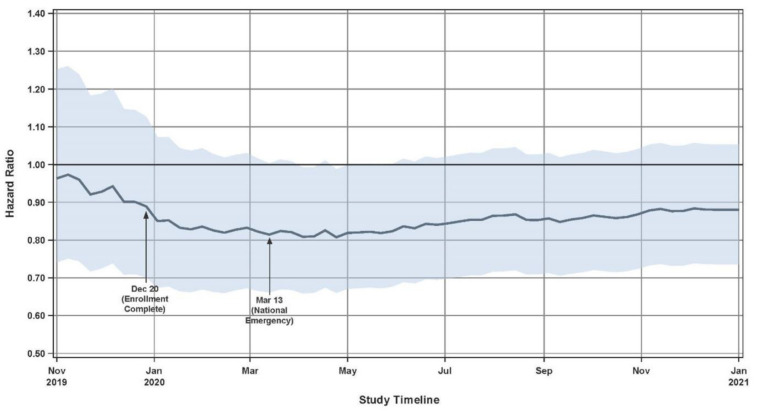
Primary endpoint hazard ratio and confidence interval evaluated over time in GUIDE-HF. Solid line reflects hazard ratio for data through each timepoint, shaded region reflects 95% confidence interval for each timepoint. Reprinted from The Lancet Volume 398, Issue 10304, Lindenfeld et al., Haemodynamic-guided management of heart failure (GUIDE-HF): a randomised controlled trial, pages 991–1001, Copyright 2021, with permission from Elsevier [28].

The second RCT was the GUIDE-HF trial [28], published five years after the completed follow-up results of CHAMPION. The rationale of GUIDE-HF was to assess whether the efficacy of the CardioMEMS HF system as shown in CHAMPION could be extrapolated to a broader range of HF patients, in this case NYHA functional class II and IV. In addition, GUIDE-HF introduced an inclusion criterion specifically for HF patients without a recent HF hospitalization, based on elevated natriuretic peptides [29]. Other inclusion and exclusion criteria were similar to CHAMPION, including optimally titrated guideline-directed medical therapy (GDMT). The primary outcome was extended to also include urgent HF visits requiring intravenous diuretic therapy, as recommended in the latest FDA guidance on cardiovascular endpoint definitions for clinical trials [30]. The follow-up was capped at twelve months. There were 253 events in the treatment group (n = 497) and 289 in the control group (n = 503), which resulted in a statistically insignificant main analysis of the primary outcome (HR 0.88; 95% CI 0.74–1.05; *p* = 0.16). While there were more changes in diuretics in the treatment group, the cumulative dose changes were unclear [31]. Implementation of GDMT at 12 months was low as compared to baseline [31]. However, this is a finding that has also been reported in real-world HF patients [32]. A COVID-19 sensitivity analysis using a time-dependent variable to compare events before and after the pandemic warranted a separate pre-COVID analysis of the primary outcome. In this pre-COVID-19 analysis, there were 177 events in the treatment group and 224 events in the control group, which yielded borderline significant results in favor of CardioMEMS (HR 0.81; 95% CI 0.66–1.00; *p* = 0.049). Influence of COVID-19 on event rates was therefore likely, as shown in Figure 2, but the overall effect was less pronounced. This was confirmed in the post-COVID-19 analysis, where the direction of effect even went in favor of the control group (HR 1.11; 95% CI 0.80–1.55; *p* = 0.53), which was explained by a 21% event rate reduction in the control group during the COVID-19 pandemic while the event rates of the treatment group remained virtually the same. The mechanism behind this finding was hypothesized to be related to reduced consumption of salt-laden processed food during lockdown, increases or decreases in exercise, better remote care, or avoidance of hospitals because of fear of COVID-19 or to protect overloaded services [31]. GUIDE-HF was criticized for enrolling a suboptimal group of patients due to many having PAPs within the target range at baseline which limits the possibility of short term gain, especially considering the short follow-up of 12 months which was also a limitation [31]. The results of GUIDE-HF were deemed encouraging but inconclusive, with a need for a large, simple, open label RCT to evaluate the system of care in real-world and not solely the technology [31].

The published CHAMPION data were convincing and formed a platform for further research, including both RCTs as well as observational studies. GUIDE-HF was a large important trial, but somewhat controversial due to the apparent COVID-19 pandemic effect which on the other hand could have been beneficial in favor of remote monitoring strategies. The rationale behind the GUIDE-HF trial was mainly to evaluate CardioMEMS effectiveness in a broader population, including patients in NYHA class II and IV, as well as patients without a HF-related hospitalization with increased NT-proBNP levels. Interestingly, in the pre-COVID-19 subgroup analysis, CardioMEMS PAP monitoring appeared beneficial in NYHA II patients, with a reported risk reduction of 34% (HR 0.66; 95% CI 0.45–0.99; *p* = 0.044), while NYHA IV patients did not experience any benefit. Despite the discussion with regard to the COVID-19 sensitivity analyses, FDA approval was acquired for patients with NYHA class II HF and patients with increased NT-proBNP levels.

#### 3.1.3. Non-Randomized Trials

The first European study investigating the CardioMEMS HF system was the post marketing MEMS-HF study, which aimed to evaluate safety, feasibility, and performance of hemodynamic-guided HF management with the CardioMEMS HF system over 12 months [33]. A total of 236 NYHA III patients with a HF-related hospitalization in the previous year were enrolled and underwent implantation of the CardioMEMS HF system. Heart failure with reduced ejection fraction (HFrEF) patients were required to receive GDMT as tolerated [33]. Physicians and non-physician caregivers were given instructions on PAP-guided care, and uploaded PAP data were reviewed at least weekly. Freedom from device- or system-related complications (DSRC) and sensor failure after 12 months were 98.3% and 99.6%, respectively [33]. Overall survival after 12 months was 86.2%. Compared to the 12 months prior to implantation of the CardioMEMS HF system, there was a reduction in HF-related hospitalizations of 62% (HR 0.38; 95% CI 0.31–0.48; *p* < 0.0001) [33]. PAP decreased with 5.0  ±  7.3 (mean, mPAP), 5.5  ±  9.3 (systolic, sPAP), and 4.6  ±  6.2 (diastolic, dPAP) mmHg after 12 months as compared to baseline (*p* < 0.0001 for all), the reductions were significantly greater in patients with a baseline mPAP ≥ 3 5. NYHA functional class was improved in 38% of the patients. Moreover, the Kansas City Cardiomyopathy Questionnaire (KCCQ) overall/clinical summary scores increased from 47.0  ±  24.0/51.2  ±  24.8 to 60.5  ±  24.3/62.4  ±  24.1 (*p*  <  0.0001), and the 9-item Patient Health Questionnaire sum score improved from 8.7  ±  5.9 to 6.3  ±  5.1 (*p*  <  0.0001) [33]. KCCQ summary score improvements ≥ 10 were associated with greater PAP decreases [33]. Most medication changes were made for diuretics, no comparison was made with the number of changes in the period prior to enrolment. The mean patient adherence to daily PAP measurements was 78.1 ± 23.5% and to weekly measurements 89.7 ± 17.8%, caregiver adherence to weekly reviews was 89.8  ±  18.7%. The MEMS-HF study thus demonstrated the CardioMEMS HF system to be safe and feasible in European setting, still comparing patients to their own historic hospitalization rate the previous year is less strong evidence, paving the way for European studies assessing the CardioMEMS HF system in randomized studies [33].

Another non-randomized study was the CardioMEMS post-approval study performed in the US, in which the objective was to assess efficacy and safety of PAP monitoring in routine clinical practice over one year, with an additional focus on previously underrepresented subgroups such as women, Blacks, patients with Heart Failure with a preserved Ejection Fraction (HFpEF), and patients with CRT or ICD [34]. Physicians received instructions on how to use PAP goals to guide therapy and to adjust medication to maintain PAP within normal range. The CardioMEMS HF system was implanted in 1200 NYHA III patients with a HF-related hospitalization within 12 months prior to inclusion and use of betablocker and angiotensin-converting enzyme inhibitor (ACEi)/angiotensin II receptor blocker (ARB) in case of HFrEF. Overall, 37.7% were female, 14.3% were Black, 19.9% had CRT, and 32.2% had an ICD. Compared with the year prior to the sensor implantation, the HF-related hospitalization rate after one year of follow-up was reduced by 57% (HR 0.43; 95% CI 0.39–0.47; *p* < 0.0001). These results were consistent across the aforementioned subgroups. After 12 months, the mPAP increased by 1.5 ± 5.8 mmHg (*p* < 0.0002) for patients with a baseline mPAP < 25 mmHg, decreased by 1.3 ± 5.0 mmHg (*p* < 0.0001) for patients with a baseline mPAP of 25–35 mmHg, and decreased by 4.8 ± 6.2 mmHg (*p* < 0.0001) for patients with a baseline mPAP ≥35 mm Hg. During the study, 81.8% of the patients had a medication change related to an increase in PAP and 55.8% had a medication change related to a decrease in PAP. Mean patient adherence to daily PAP measurements was 76 ± 24% and to weekly PAP measurements 93 ± 16%. Freedom from DSRC and sensor failure were 99.6% and 99.9%, respectively. Recently, the extended follow-up results of the post-approval study were also published [35], with 710 patients completing 24 months of follow-up. Compared to the pre-implant period, there was a HF-related hospitalization rate reduction of 70% (HR 0.30; 95% CI 0.25–0.35; *p* < 0.0001) after two years of follow-up. Moreover, compared to the one year follow-up, the two year follow-up had a reduction in HF-related hospitalizations of 38% (HR 0.62; 95% CI 0.56–0.69; *p* < 0.0001). The mPAP decreased from 34.3 ± 10.2 mmHg during the first week of follow-up to 30.3 ± 9.1 mmHg after one year and 29.7 ± 9.3 mmHg after two years (*p* < 0.0001). Freedom from DSRC and sensor failure after two years was 99.6% and 99.9%, respectively. The findings from this study therefore confirmed the generalizability of previous results to underrepresented subgroups. Still, the comparison in this study is the hospitalization rate the year pre-implant (historical control) versus the year post implant which provides less strong scientific evidence due to lack of randomization and lack of a comparator of usual care.

**Figure 3:**
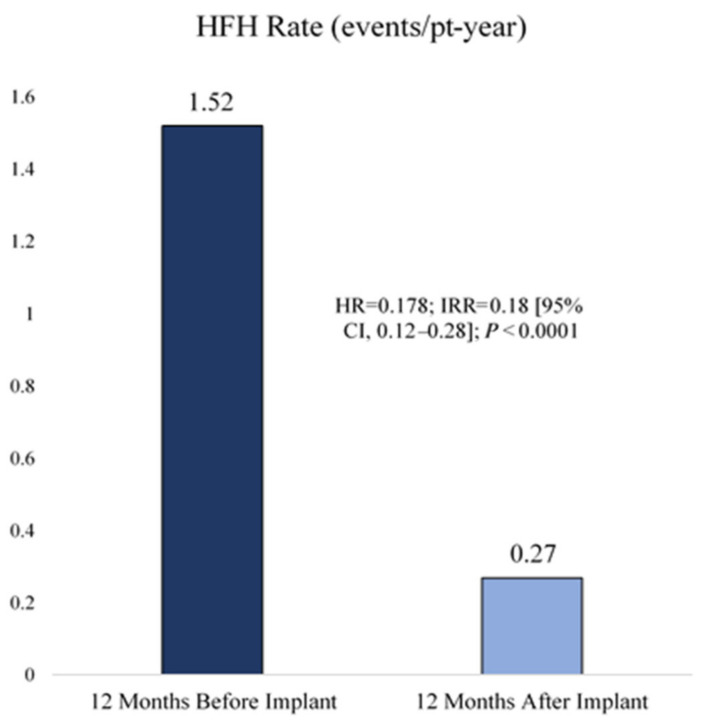
Heart failure-related hospitalization reduction in COAST. HFH: Heart failure hospitalization (HFH) reduction. Reprinted from ESC Heart Failure Volume 9, Issue 1, Cowie et al., Real-world evidence in a national health service: results of the UK CardioMEMS HF System Post-Market Study, pages 48–56, Copyright 2022, with permission from Elsevier [36].

Finally, the (post-marketing) non-randomized single arm study COAST was performed in the United Kingdom (UK), Europe, and Australia [23], of which only the UK results have yet been published in a short communication [36]. The goal was again to assess feasibility, safety, and clinical benefit of PAP monitoring as hemodynamic-guided management strategy, but this time in more diverse geographic settings [36]. A total of 100 patients with NYHA III, a HF-related hospitalization within twelve months prior to inclusion, and use of betablocker and ACEi/ARB for HFrEF had a CardioMEMS HF system implanted and were followed for two years. The design of COAST was similar to the previous two non-randomized studies, in that the HF-related hospitalization rate in the twelve months prior to inclusion was compared to the period after inclusion (historic own control group). The hospitalization rate after implantation was 82% lower as compared to the twelve months prior to enrolment (HR 0.18; 95% CI 0.12–0.28; *p* < 0.0001) as shown in Figure 3. Freedom from DSRC and sensor failure after two years were 100% and 99%, respectively. PAP decreased with 3.3 ± 4.5 (mPAP), 4.2 ± 6.6 (sPAP), and 2.7 ± 3.7 (dPAP) mmHg after 12 months as compared to baseline (*p* < 0.0001 for all). The EQ-5D-5L quality of life questionnaire and index scores remained stable during the study. The mean visual analogue scale score component at twelve months improved by 1.9 ± 20 (95% CI −3.0–6.9; *p* = 0.4344) compared to baseline, but was not a significant change. The NYHA functional class also improved after 12 months with 43% of the initial NYHA III patients transferring to NYHA I and II. Most medication changes were made for diuretics; however, no comparison was made with the number of changes in the period prior to enrolment. COAST reported results which were in accordance with earlier studies and confirmed that PAP monitoring as hemodynamic-guided management strategy is feasible, safe, and appeared to be effective in the healthcare setting of the UK.

Additionally, two smaller studies with respectively 11 and 5 patients in Spain and Portugal also reported positive results, but were reported mainly as first experiences in these countries [37,38].

Findings from the non-randomized studies are in line with the RCTs [36,39]. However, it is important to carefully interpret efficacy findings from these studies as they were non-randomized and are prone to important biases. Randomized clinical trials are the golden standard for the assessment of clinical efficacy.

#### 3.1.4. Cohort Studies

While effectiveness of PAP monitoring with the CardioMEMS HF system was shown in multiple RCTs, the association between this hemodynamic-guided management strategy and clinical outcomes in real-world clinical practice was not yet known. The goal of one of the first cohort studies using the CardioMEMS HF system was to provide an answer to this question [40]. In this study, patients who underwent implantation of the CardioMEMS HF system were retrospectively identified using insurance data. This cohort was then filtered on patients with follow-up data 6 months before and after sensor implantation. Additionally, patients with follow-up data 12 months before and after sensor implantation were filtered for a separate analysis. The periods before and after implantation were then compared on HF-related hospitalization rates. The resulting cohorts comprised of 1114 patients for the 6 months analysis and 480 patients for the 12 months analysis. In the 6 months analysis, 1020 HF-related hospitalizations occurred before and 381 after sensor implantation, the HF-hospitalization rate was reduced by 45% after implantation (HR 0.55; 95% CI 0.49–0.61; *p* < 0.001). In the 12 months analysis, 696 HF-related hospitalizations occurred in the pre-implant period as compared to 300 HF-related hospitalizations in the post-implant period, the HF-hospitalization rate was reduced by 34% (HR 0.66; 95% CI 0.57–0.76; *p* < 0.001). These results from a non-trial population compare favorably with the results from the RCTs and offer confidence in the effectiveness of PAP monitoring with the CardioMEMS HF system in real-world clinical practice.

Two years later, another observational study assessing CardioMEMS with insurance data was performed [41], but this time with a comparator arm and longer mean/median follow-up time. While RCTs remain the gold standard in obtaining unbiased results from comparisons between treatment and control groups, advanced statistical techniques, can be used to account for certain biases. In this study, patients who underwent implantation of a CardioMEMS HF system (treatment cohort) were retrospectively identified using US insurance data and were compared with patients who did not receive a sensor in a matched cohort analysis. The treatment cohort was filtered for patients who had a HF-related hospitalization in the 12 months prior to sensor implantation and were followed for 12 months after sensor implantation. This resulted in 1087 treatment and control pairs that were matched on demographic attributes, comorbidities, and propensity scores to obtain exchangeability. Twelve months after sensor implantation, 616 HF-related hospitalizations occurred in the treatment cohort and 784 in the control cohort, and the HF-hospitalization rate was 24% lower in the treatment cohort (HR 0.76; 95% CI 0.65–0.89; *p* < 0.01).

**Figure 4:**
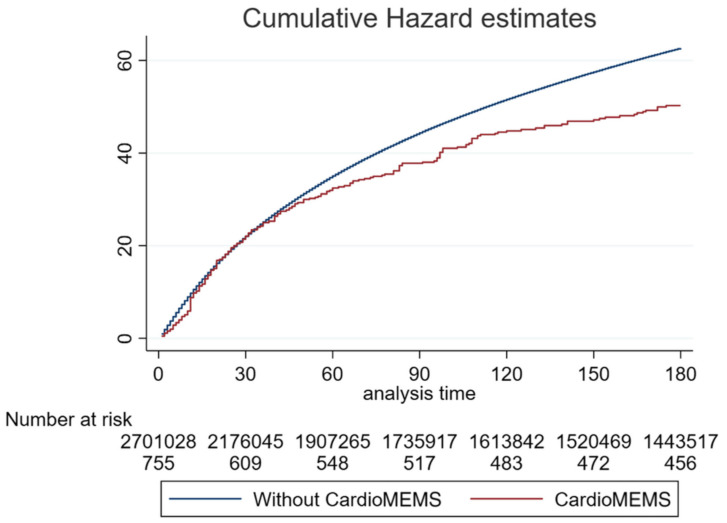
Cumulative incidence of heart failure readmissions during 180 days after discharge, stratified ac-cording to treatment (without or with CardioMEMS). Reprinted from ESC Heart Failure Volume 9, Issue 4, Kishino et al., Effect of pulmonary artery pressure-guided therapy on heart failure read-mission in a nationally representative cohort, pages 2511–2517, Copyright 2022, with permission from Elsevier [42].

Finally, another more recent study was performed in the US where the Nationwide Readmissions Database (NRD) was used to evaluate the clinical benefit of the CardioMEMS HF system [42]. This study included patients with an implanted CardioMEMS HF system that were hospitalized for acute HF within a five year time window and compared them to a control cohort on HF rehospitalization rates at 30, 90, and 180 days. For the comparison at 30 days, a propensity score matched cohort was used. Before matching, 1842 HF patients with an implanted CardioMEMS HF system and 5,326,530 HF patients without were identified. After propensity score matching, 1839 HF patients with a sensor and 1924 without remained. In the unmatched cohort, rehospitalization occurred 17.3% versus 20.9% (*p* = 0.02) at 30 days, 29.6% versus 36.5% at 90 days (*p* = 0.002), and 39.6% versus 46.6% at 180 days (*p* = 0.009) in patients with versus without the CardioMEMS HF system (Figure 4). In the 30-day analysis of the matched cohort, the difference in rehospitalization remained significant and occurred 17.3% versus 21.5% (*p* = 0.02) in patients with versus without the CardioMEMS HF system. In multivariable regression analysis, the CardioMEMS HF system reduced rehospitalization rates by 25% (HR 0.75; 95% CI 0.63–0.89; *p* = 0.001) at 30 days, 27% (HR 0.73; 95% CI 0.63–0.86; *p* < 0.001) at 90 days, and 20% (HR 0.80; 95% CI 0.71–0.91; *p* = 0.001) at 180 days.

Additionally, a smaller cohort study was done with the Veterans Affairs Healthcare System where 53 patients with an implanted CardioMEMS HF system were retrospectively identified [43]. Similar to earlier cohort studies, HF-related hospitalization rates in the period 6 and 12 months before, and 6 and 12 months after implantation were compared. In the 6 months analysis, the HF-hospitalization incidence rate was reduced by 52% (Incidence rate ratio (IRR) 0.48; 95% CI 0.32–0.70; *p* < 0.001) and by 44% in the 12 months analysis (IRR 0.56; 0.41–0.76; *p* < 0.001). The mPAP decreased from 33.4 ± 10.9 mmHg at baseline to 30.2 ± 9.6 mmHg at 6 months (*p* = 0.035) and decreased further to 28.3 ± 8.8 mmHg (*p* = 0.007) at 12 months, whereas the sPAP decreased from 51.2 ± 16.8 mmHg at baseline to 44.2 ± 12.6 mmHg at 6 months (*p* = 0.001), and to 41.7 ± 11.7 mmHg at 12 months (*p* < 0.001). The dPAP decreased from 23.6 ± 8.2 mmHg at baseline to 21.2 ± 8.1 mmHg at 6 months (*p* = 0.076) and to 20.5 ± 7.5 mmHg at 12 months (*p* = 0.09). Most medication changes were made to diuretics, with 81% of the patients having their diuretics adjusted.

In brief, results from real-world studies as discussed above are in line with the RCTs and observational studies, which strengthens the evidence base for CardioMEMS and further supports its usefulness in daily clinical practice outside of a study setting.

#### 3.1.5. Clinically Relevant Subanalyses

Several subanalyses from the aforementioned studies have been performed. Based on CHAMPION, subanalyses have been performed to assess effectiveness of the CardioMEMS HF system in patients with heart failure with preserved ejection fraction (HFpEF) [18], patients with HFrEF [19], patients with left ventricular assist device implantation [44], and patients with CRT [45]. Significant benefit of the CardioMEMS HF system was shown in all these subgroups, in line with the main analysis. Additionally, another CHAMPION subanalysis with an average additional follow-up of thirteen months on top of the RCT results showed that PAP-guided HF management reduced the HF-related hospitalization rate by 49% (HR 0.51; 95% CI 0.37–0.70; *p* < 0.0001) and 30-day all-cause readmission rate by 58% (HR 0.42; 95% CI 0.22–0.80; *p* = 0.0080) [46].

Furthermore, two newer subanalyses on sacubitril/valsartan (Angiotensin Receptor-Neprilysin Inhibitor, ARNi) use and subtypes of pulmonary hypertension were performed with data from the MEMS-HF study [47,48]. The first subanalysis showed an association between ARNi use and reduced utilization of loop diuretics in patients with PAP-guided treatment. The second subanalysis showed significant benefit of remote PAP monitoring in patients with pulmonary hypertension (PH), regardless of the PH subtype.

Finally, based on the CardioMEMS post-approval study, subanalyses for sex and obesity were performed [49,50]. In the sex subanalysis, similar reductions in HF-related hospitalizations were seen in males and females with a 54% reduction (HR 0.46; 95% CI 0.40–0.52; *p* < 0.001) for males, and a 61% reduction (HR 0.39; 95% CI 0.33–0.46; *p* < 0.001) for females, which is reassuring considering the differences in HF treatment by sex [51]. PAP reductions were also similar in males and females. In the obesity subanalysis, similar reductions in HF-related hospitalizations regardless of ejection fraction were also found in obese (BMI ≥ 35 kg/m^2^) and non-obese (BMI < 35 kg/m^2^) patients, with reductions of 63% (HR 0.37; 95% CI 0.30–0.45; *p* < 0.0001) and 55% (HR 0.45; 95% CI 0.40–0.51; *p* < 0.0001), respectively.

The CHAMPION subanalysis for patients with HFpEF is interesting as effective strategies for HFpEF are scarce, implying potential for CardioMEMS in this relevant patient group. Furthermore, usefulness in pulmonary hypertension as confirmed by the MEMS HF investigators is relevant, as PH is often encountered in clinical practice. The subgroup analyses from the post-approval study, the largest observational study to date, are especially reassuring considering the vast underrepresentation of these subgroups in clinical trials.

### 3.2. Cordella HF System

#### 3.2.1. Device Information

**Figure 5:**
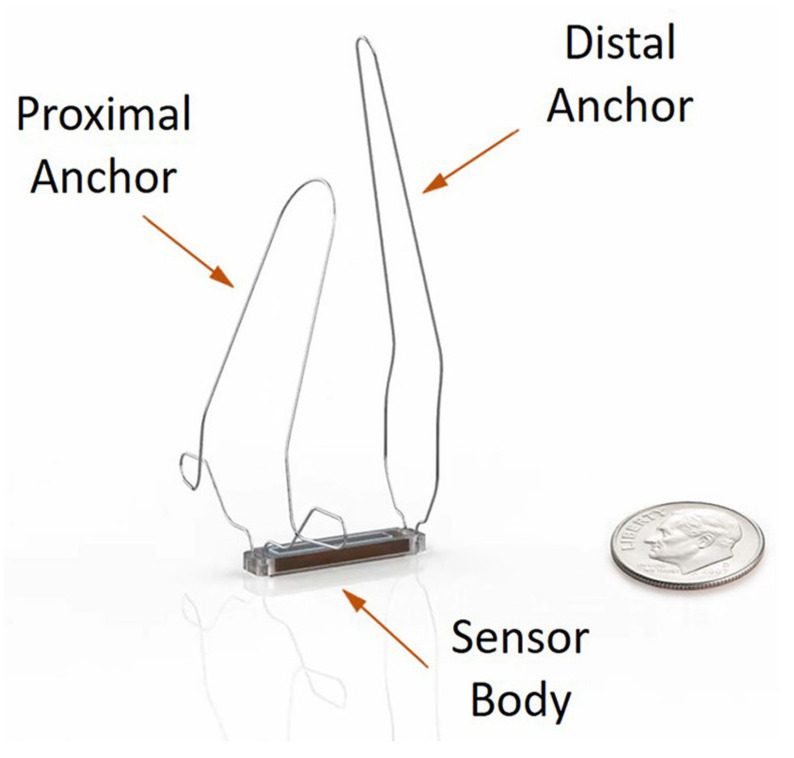
The Cordella HF system pulmonary artery pressure sensor. Reprinted from European Journal of Heart Failure Volume 22, Issue 10, Mullens et al., Effect of pulmonary artery pressure-guided therapy on heart failure readmission in a nationally representative cohort, pages 1912–1919, Creative Commons CC BY license [52].

The Cordella HF system includes a PAP sensor and also uses contact with the patient for daily vital signs such as blood pressure, heart rate, weight, and oxygen saturations (Figure 5) [52]. Similar to the CardioMEMS HF system, the Cordella PAP sensor is permanently implanted, but in the right pulmonary artery. Part of the system is a handheld reader in the form of a tablet. Patients are instructed to take daily readings, although additional readings can be done if necessary. These daily readings are performed by undocking the measurement device, holding it against the chest with one hand while seated, and redocking the device afterwards.

#### 3.2.2. Non-Randomized Trials

**Figure 6:**
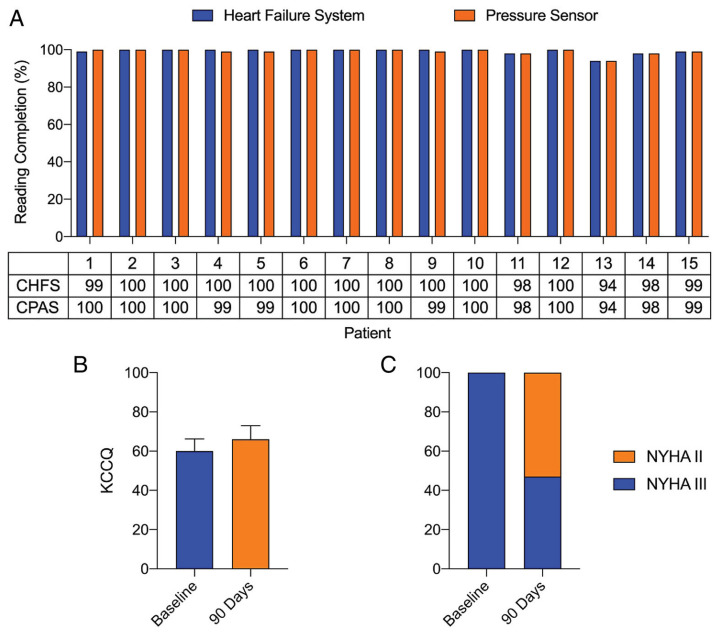
Summary of the results from the SIRONA study. Patient adherence, quality of life and New York Heart Association (NYHA) classification. (**A**) Patient adherence with daily Cordella Heart Failure System (CHFS) and Cordella Pulmonary Artery Pressure Sensor (CPAS) readings over the first 90 days following implantation. (**B**) Kansas City Cardiomyopathy Questionnaire (KCCQ) score at baseline and 90 days following implantation. (**C**) NYHA classification at baseline and 90 days following implantation. Reprinted from European Journal of Heart Failure Volume 22, Issue 10, Mullens et al., Digital health care solution for proactive heart failure management with the Cordella Heart Failure System: results of the SIRONA first-in-human study, pages 1912–1919, Creative Commons CC BY license [52].

The first study that assessed the new Cordella HF System technology was the SIRONA first-in-human study [52]. The goal of this study was to examine the safety, accuracy, and usability of the Cordella HF system. Fifteen NYHA III HF patients with a HF-related hospitalization in the previous 12 months were included and underwent implantation of the Cordella HF System PAP sensor. Physicians were blinded to the PAP readings for the first 90 days, after which the accuracy of the Cordella HF system PAP sensor was assessed with right heart catheterization. The primary safety endpoint was freedom from DSRC in the first 30 days and sensor failure. The primary efficacy endpoint was accuracy of the Cordella PAP measurements compared to a fluid-filled catheter. Secondary efficacy endpoints included change in PAP after implantation, frequency of HF-related hospitalizations, change in quality of life, and patient adherence to the daily measurements. After 90 days, four adverse events related to the Cordella PAP sensor were reported, but no DSRC causing invasive treatment, device explant, or death occurred. Freedom from sensor failure after 90 days was 100% and one HF-related hospitalization occurred. The measured PAP values of the Cordella PAP sensor were well matched with the values measured by right heart catheterization with an average mPAP of 22.5 ±11.8 mmHg and 25.2 ± 8.5 mmHg, respectively. Patient adherence to the daily readings was 99% and there was improvement in quality of life and NYHA functional class. A summary of the results of the SIRONA study is included in Figure 6.

**Figure 7:**
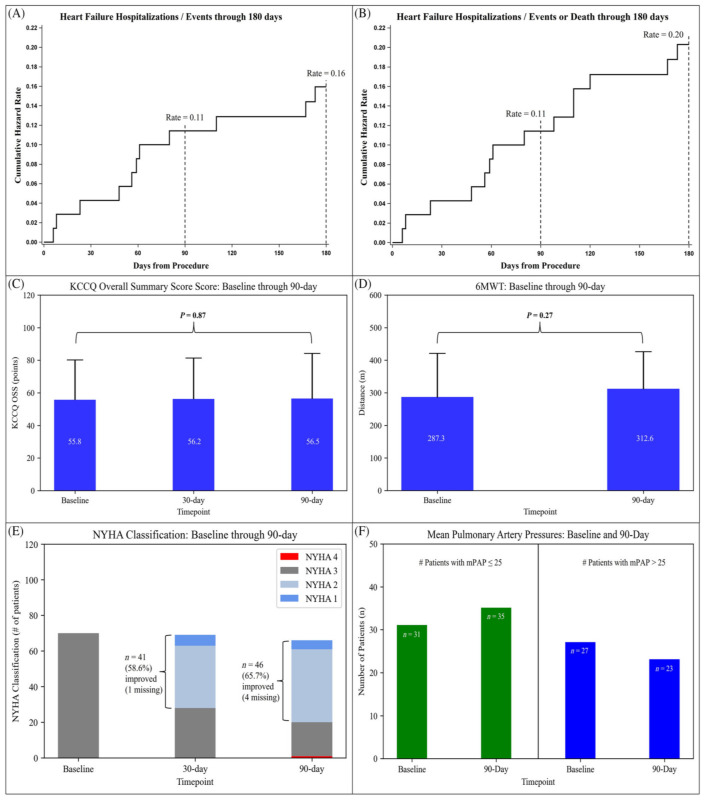
Summary of the results of the SIRONA 2 study. (**A**) Cumulative hazard rate curve through 180 days for HF hospitalizations, HF treatments in a hospital day-care setting, or urgent outpatient clinic HF visits. (**B**) Cumulative hazard rate curve through 180 days for HF hospitalizations, HF treatments in a hospital day-care setting, or urgent outpatient clinic HF visits and death. (**C**) Baseline, 1, 3 month KCCQ Overall Summary Score. (**D**) Baseline, 3 month 6 MWT. (**E**) Baseline, 1, 3 month NYHA Classification. (**F**) Number of patients with mean pulmonary artery pressure (mPAP) ≤ 25 and mPAP > 25 mmHg at both baseline and 90 days. Reprinted from ESC Heart Failure Volume 9, Issue 5, Sharif et al., Safety and efficacy of a wireless pulmonary artery pressure sensor: primary endpoint results of the SIRONA 2 clinical trial, pages 2862–2872, Copyright 2022, with permission from Elsevier [21].

The larger SIRONA 2 trial was the successor of the SIRONA trial and also aimed to evaluate safety and efficacy of the Cordella HF System [21]. A total of 70 NYHA III HF patients with a HF-related hospitalization or elevated NT-proBNP in the previous twelve months were included. The design was similar to the SIRONA trial, with the primary safety endpoint being freedom from DSRC and sensor failure and the primary efficacy endpoint being the accuracy of the PAP sensor measurements compared to fluid-filled catheter measurements after 90 days. Secondary efficacy endpoints included change in PAP, frequency of HF-related hospitalizations, quality of life and functional changes, and patient adherence to daily readings. The sensor was implanted successfully during the first attempt in all patients. There were a total of six adverse events and two serious adverse events after 30 days, of which one was adjudicated as DSRC, and there were no sensor failures. The right heart catheterization after 90 days showed good accuracy of the Cordella PAP sensor, with average mPAP values of 27.6 ± 12.1 mmHg and 26.1 ± 10.4 mmHg for the Cordella PAP sensor and fluid-filled catheter, respectively. At baseline, 53.4% of the patients had a mPAP ≤ 25 mmHg and 46.6% > 25 mmHg, which transitioned into 60.3% with a mPAP ≤ 25 mmHg and 39.7% > 25 mmHg after 90 days. A total of 8 patients suffered a HF-related hospitalization after 90 days of follow-up, and 11 patients did so after 6 months of follow-up. Quality of life scores remained unchanged after 90 days compared to baseline, while the NYHA functional class improved in 65.7% of the patients. Patient adherence was high with 95% taking readings more than 5 out of 7 days after one month, 94% after 3 months, and 93% after 6 months. A summary of the results of the SIRONA II study are included in Figure 7.

Both SIRONA trials showed good safety, accuracy, and feasibility of the Cordella PAP sensor, making it a promising technology for PAP-guided management of HF patients. However, randomized clinical trials comparing the Cordella HF system with standard care are needed to confirm clinical benefit. Another limitation was the small sample size of the studies, which also limited statistical power for relevant clinical outcomes.

#### 3.2.3. Cohort Studies

**Figure 8:**
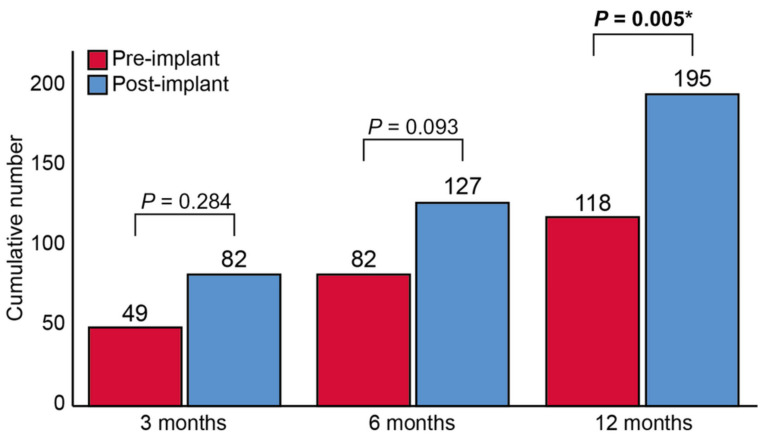
Total number of diuretic changes pre-implantation and post-implantation. Total number of diuretic changes pre-implantation and post-implantation. * *p* < 0.05. Reprinted from ESC Heart Failure Volume 9, Issue 6, Dauw et al., Ambulatory haemodynamic-guided management reduces heart failure hospitalizations in a multicentre European heart failure cohort, pages 3858–3867, Copyright 2022, with permission from Elsevier [53].

The only cohort study including the Cordella HF system was a study that assessed PAP-guided management with both the CardioMEMS and Cordella Systems [53]. A total of 48 HF patients (29 with CardioMEMS and 19 with Cordella devices) were included and the number of diuretic changes, HF-related hospitalizations, and HF-related costs were compared between 3, 6, and 12 months before and after sensor implantation. Additionally, the change in PAP between baseline and 1 year follow-up was assessed. For the HF-related hospitalizations, there was a non-significant difference between the pre- and post-implant period after 3 months with 22 versus 12 HF-related hospitalizations (*p* = 0.098). After 6 and 12 months, there was a significant difference with 34 versus 17 HF-related hospitalizations (*p* = 0.014), and 48 versus 29 HF-related hospitalizations (*p* = 0.032), respectively. For diuretic changes, there was a non-significant difference in the pre-implant versus post-implant period for 3 and 6 months with 49 versus 82 changes (*p* = 0.284) and 82 versus 127 changes (*p* = 0.093), respectively. However, for 12 months there was a significant difference with 118 versus 195 changes (*p* = 0.005) (Figure 8). There were very few changes to other HF drugs. For patients with a baseline mPAP < 25 mmHg, the mPAP was similar between baseline and at one year follow-up with 16 ± 6 versus 19 ± 13 mmHg (*p* = 0.127). For patients with a baseline mPAP ≥ 25 mmHg, the mPAP decreased from 40 ± 9 mmHg at baseline to 34 ± 11 mmHg after 1 year (*p* = 0.085). Patient compliance to daily PAP measurements was 87 ± 20% after 1 month and 66 ± 32% after 1 year. This was 99 ± 6% and 94 ± 11% for the compliance to weekly measurements. Again, the small sample size limited the interpretation of these findings.

### 3.3. Pulmonary Artery Pressure Monitoring to Assess Medical Therapy Effects

Pulmonary artery pressure monitoring has also been used to study the effects of HF drugs on PAP. One of these studies retrospectively identified 18 HFrEF patients with an implanted CardioMEMS HF system and assessed the effect of ARNi on PA pressures, by comparing averaged pressure data 1 to 5 days before and after initiation [54]. The transition from ACEi or ARB to ARNi was associated with a significant reduction of PAP. The median reduction was −3.6 (IQR −9.8–−0.7) mmHg for mPAP (*p* < 0.001), −6.5 (IQR −15.0–−2.0) mmHg for sPAP (*p* = 0.001), and −2.5 (IQR −5.7–−0.7) for dPAP (*p* = 0.001), which is interesting from a pathophysiological point of view.

A similar study also investigated the effects of ARNi on PAP in a prospective cohort of 13 HFrEF patients with an implanted sensor [55]. Compared to one week prior to initiation, PA pressures one week after imitation were significantly lower with a mean reduction of 3.2 ± 3.9 mmHg for mPAP (*p* = 0.015), 3.6 ± 4.9 mmHg for sPAP (*p* = 0.027), and 2.5 ± 3.1 mmHg for dPAP (*p* = 0.020).

Finally, a recent study assessed the effect of ARNi on PA pressure in 14 HFpEF patients with an implanted sensor [56]. Daily PAP measurements were studied during three periods: six weeks prior to initiation of ARNi, six weeks after initiation, and six weeks after withdrawal. Compared to the period prior to initiation, the mPAP significantly decreased by −4.99 (95% CI −5.55–−4.43) mmHg after the ARNi was introduced. After withdrawal of the ARNi, the mPAP significantly increased by 2.84 (95% CI 2.26–3.42) mmHg. These studies show the clear beneficial hemodynamic effects of ARNi in both HFrEF and HFpEF patients.

A comparable study has also been performed for sodium-glucose cotransporter 2 inhibitors (SGLT2i) [57], which is a novel addition to GDMT for HFrEF patients and has also shown benefit for HFpEF patients [58,59,60,61]. In this study, HFrEF patients with either a CardioMEMS HF system or Cordella PAP Sensor were included. Daily PAP measurements seven days before and after SGLT2i initiation were compared, and showed that addition of SGLT2 resulted in a significant decrease in average mPAP from 42 ± 9.16 to 38 ± 9.95 mmHg (*p* < 0.05).

These studies showed that PAP monitoring also enables the unique possibility to study hemodynamic effects of medical therapy in detail, which is interesting from a physician’s perspective and has not yet been possible on continuous basis in ambulatory setting. As the volume of patients with a PAP sensor will gradually increase, knowledge on the hemodynamic effects of HF drugs, including the newest drugs, is likely to expand in the near future.

## 4. Ongoing Studies

### 4.1. CardioMEMS HF System

The MONITOR HF trial is an ongoing RCT of which the results are greatly anticipated [39]. After the results of CHAMPION and GUIDE-HF, there was a need for a European RCT. The results of MONITOR HF are especially interesting because the results of GUIDE-HF were not fully conclusive since the results of the main analysis did not show a significant effect, while there was a significant effect in the COVID-19 analysis [28]. Moreover, although European single arm studies showed positive results, European RCT data are not yet available but urgently needed to assess the effectiveness of the CardioMEMS HF system. The MONITOR HF trial will therefore provide valuable data to demonstrate effectiveness of the CardioMEMS HF system in an European healthcare setting, which differs significantly from the US in structure and organization. With the high level of standard care in the Netherlands, as shown in the CHECK-HF registry compared to the CHAMP HF registry [62,63], the results of this RCT will be very generalizable to other western European countries such as Belgium, France, the UK, and Germany. This trial will also focus on cost-effectiveness, with detailed information on treatment changes, medical care consumption, and quality of life. The results will therefore also play a major role in the process of reimbursement of the CardioMEMS HF system in Europe. In this RCT, 340 NYHA III patients will be randomized 1:1 to receive either standard care or hemodynamic guided care using the CardioMEMS HF system on top of standard care. Eligible patients need to have had a HF-related hospitalization in the twelve months prior to inclusion and HFrEF patients needed to be on optimized GDMT. The minimum follow-up for all patients will be one year [39]. Results are expected in the first half of 2023.

Finally, PASSPORT-HF, another RCT assessing the CardioMEMS HF system, was recently announced [64]. This trial will take place in Germany, another country with a high-quality healthcare system. The design and goal of this RCT are similar to the MONITOR HF trial, with the exception of structured telephone contacts that are implemented in both study arms and the primary outcome being a composite of all-cause mortality and HF-related hospitalizations. PASSPORT-HF aims to enroll 554 NYHA III patients and, similar to MONITOR HF, with the same HF-related hospitalization and GDMT requirements and a follow-up of twelve months. Considering the resources that are needed to conduct an RCT, the question arises what the added value of PASSPORT-HF will be as compared to the earlier started MONITOR HF trial, especially since the healthcare system of the Netherlands is relatively generalizable to other western European countries. Results are expected after 2024.

### 4.2. Cordella PAP Sensor

The PROACTIVE-HF trial is a single-arm trial investigating the Cordella System with prespecified safety and effectiveness endpoints. The trial is currently enrolling patients in sites across the US and Belgium, and was initially designed as multicenter randomized clinical trial. Its design was comparable to earlier RCTs from the US as all enrolled patients had the Cordella sensor implanted and were then randomized to a treatment or control group in a 1:1 ratio. All patients were instructed to perform measurements of PA pressures and vital signs on a daily basis. These data were available to the healthcare providers of the treatment arm, but the patients in the treatment arm only had access to their vital signs, not to the PAP data. Only the vital signs were available for the patients and healthcare providers in the control group. Unblinding to PAP data was done after 12 months. Enrollment criteria were comparable to the earlier trials. In brief, adult HF patients, regardless of LVEF, in NYHA functional class III with either a HF-related hospitalization or persistently elevated NT-proBNP levels, who were treated with optimal doses of GDMT for a minimum of 3 months, were eligible for inclusion. An important aspect of this trial is the treatment guideline or management strategy proposed by the investigators to optimize and maintain GDMT. Furthermore, a treatment algorithm mainly consisting of diuretics, is provided to lower PA pressures. The investigators hypothesized that the information on vital signs may result in medication changes in favor of GDMT. Importantly, the design of PROACTIVE-HF was changed, partially due to emerging evidence for the beneficial effects of PAP-guided management. In December 2021, the design was changed into a single-arm trial with prespecified effectiveness and safety endpoints, abandoning the randomized comparison of treatment arms. Moreover, sites outside the US were included for enrollment (such as Belgium). These changes were done following consultation with the FDA. The goal was to provide evidence of a similar risk/benefit profile to the CardioMEMS HF system. After the change in design, PAP data were made available to clinicians and patients in the former control group. Importantly, these former control patients will be excluded from the effectiveness endpoint (n = 76). The patients in the former treatment group as well as the newly enrolled patients will be analyzed for the safety and effectiveness endpoints.

In order to assess effectiveness, results from CardioMEMS studies and a meta-analysis of CHAMPION, the post-approval study, MEMS-HF, GUIDE-HF, and LAPTOP-HF were used to define a performance goal (PG) [24,28,33,65]. Furthermore, the observed event rate in PROACTIVE-HF needs to be lower than the lowest observed event rates of CardioMEMS control patients, which was also adjusted further down to account for the potential impact of SGLT2i. As a result, for the primary endpoint of HF-hospitalization and all-cause mortality at six months, a PG of 0.43 events per patient per 6-months was selected, and the observed event rate must be lower than 0.37. The null hypothesis will be rejected if the upper confidence bound for the event rate in the trial is lower than this PG. The primary safety endpoints are freedom from DSRC and sensor failure. Results are expected early 2024.

Unfortunately, PROACTIVE-HF will not provide randomized results due to the design change, which limits assessment of definitive efficacy.

## 5. Differences between the Pulmonary Artery Pressure Monitoring Systems

While the underlying technique of the PAP measurements is similar in both the CardioMEMS and Cordella HF systems [66], there are differences between the two devices. The Cordella HF system combines PAP measurements with vital parameters, such as blood pressure, heart rate, weight, and oxygen saturations whereas the CardioMEMS HF system relies on PAP measurements only. However, it should be noted that vital parameters have been shown to be poorly correlated with filling pressures [12].

The implantation procedure of the two PAP sensors also differs, as the Cordella HF system sensor is implanted in the right pulmonary artery and the CardioMEMS HF system sensor in the left pulmonary artery. Moreover, the CardioMEMS HF system has a measurement device in the form of a pillow while the Cordella HF system uses a measurement device with a docking station and a tablet which allows patients to see their own measurements.

Finally, the main difference is the available evidence as shown in Table 1**,** as multiple studies have assessed the effect of the CardioMEMS HF system on HF-related hospitalizations with a comparator arm, while this has not yet been done for the Cordella HF system. In essence, we do not expect different reults.

## 6. Conclusions and Future Perspectives

This review aimed to provide a comprehensive overview of both conducted and ongoing studies on remote PAP monitoring. PAP monitoring has shown great promises with a clinically useful and intuitive parameter to monitor. Current evidence shows remote PAP monitoring to be safe, effective, and feasible, especially in the US healthcare system. Considering the clear differences between the US and European healthcare systems and quality of and access to care, it is important to have more geographic diverse randomized data from the CardioMEMS HF system against the background of a true contemporary and high standard of care comparison group.

If proven effective, an important factor in the added benefit of remote PAP monitoring is the integration in regular HF care. Healthcare providers should be trained in how to use daily PAP readings to timely intervene and avoid patient decompensation and hospitalization. Furthermore, time and resources should be allocated to give remote PAP monitoring a structural place in routine care instead of only in the context of PAP monitoring studies. Both patient and physician adherence are also indispensable for this technology to work, as the effectiveness is mediated through proactive actions of the healthcare provider based on data obtained from the patient.

A limitation of PAP monitoring is that without integration into regular HF care, which requires too much time and resources of the physicians, the monitoring tool will likely not work. The PAP measurements themselves are merely diagnostic and will only provide the physician with proxy data on the filling pressures. It is up to the physicians to proactively intervene in order to truly achieve benefit for the patients in terms of avoiding HF-related hospitalizations. Finally, another limitation is the need for an invasive procedure to implant the PAP sensor. While the safety outcomes of the studies were favorable, the complication rate is not zero.

With the increasing need for remote care due to the high burden of HF on hospital resources, PAP monitoring can become an important part of integrated HF care in the near future with further implementation and upscaling of remote monitoring techniques.

## Figures and Tables

**Table 1:** Overview of the main studies assessing pulmonary artery pressure monitoring for heart failure.

First Author	Year	PAP Monitoring Device	Study Design	Study Name	Study Population	Sample Size	Comparator	HFH Outcomes
Abraham	2011	CardioMEMS	RCT	CHAMPION	NYHA III HF and HFH < 12 months	550	Control group	HR 0.72; 95% CI 0.60–0.85; *p* = 0.0002 (6 months follow-up) HR 0.63; 95% CI 0.52–0.77; *p* < 0.0001) (15 months mean follow-up)
Abraham	2016	CardioMEMS	RCT	CHAMPION (extended follow-up)	NYHA III HF and HFH < 12 months	347	Control group and patient own historic control	HR 0.67; 95% CI 0.55–0.80; *p* < 0.0001 (18 months mean follow-up, compared to control group) HR 0.52; 95% CI 0.40–0.69; *p* < 0.0001 (13 months mean follow-up, compared to historic control)
Lindenfeld	2021	CardioMEMS	RCT	GUIDE-HF	NYHA II–IV HF and HFH < 12 months or elevated NT-proBNP/BNP	1000	Control group	HR 0.88; 95% CI 0.74–1.05; *p* = 0.16 (main analysis) HR 0.81; 95% CI 0.66–1.00; *p* = 0.049 (pre-COVID analysis)
Angermann	2020	CardioMEMS	Non-randomized trial and Post-marketing	MEMS-HF	NYHA III HF and HFH < 12 months	236	Patient own historic control	HR 0.38; 95% CI 0.31–0.48; *p* < 0.0001 (12 months post-implant versus 12 months pre-implant)
Shavelle	2020	CardioMEMS	Non-randomized trial and Post-marketing	CardioMEMS post-approval study	NYHA III HF and HFH < 12 months	1200	Patient own historic control	HR 0.43; 95% CI 0.39–0.47; *p* < 0.0001 (12 months post-implant versus 12 months pre-implant)
Heywood	2022	CardioMEMS	Non-randomized trial and Post-marketing	CardioMEMS post-approval study (extended follow-up)	NYHA III HF and HFH < 12 months	710	Patient own historic control	HR 0.30; 95% CI 0.25–0.35; *p* < 0.0001 (24 months post-implant versus 12 months pre-implant HR 0.62; 95% CI 0.56–0.69; *p* < 0.0001 (24 months post-implant versus 12 months post-implant)
Cowie	2022	CardioMEMS	Non-randomized trial	COAST (UK results)	NYHA III HF and HFH < 12 months	100	Patient own historic control	HR 0.18; 95% CI 0.12–0.28; *p* < 0.0001 (12 months post-implant versus 12 months pre-implant)
Desai	2017	CardioMEMS	Cohort study	…	HF patients with an implanted CardioMEMS and ≥6 months follow-up	1114	Patient own historic control	HR 0.55; 95% CI 0.49–0.61; *p* < 0.001 (6 months post-implant versus 6 months pre-implant) HR 0.66; 95% CI 0.57–0.76; *p* < 0.001 (12 months post-implant versus 12 months pre-implant)
Abraham	2019	CardioMEMS	Cohort study	…	HF patients with an implanted CardioMEMS and >12 months follow-up	2174	Matched control group	HR 0.76; 95% CI 0.65–0.89; *p* < 0.01 (12 months follow-up)
Kishino	2022	CardioMEMS	Cohort study	…	HF patients with an implanted CardioMEMS AND an HFH	3763	Matched control group	HR 0.75; 95% CI 0.63–0.89; *p* = 0.001 (30 days follow-up) HR 0.73; 95% CI 0.63–0.86 *p* < 0.001 (90 days follow-up) HR 0.80; 95% CI 0.71–0.91; *p* = 0.001 (180 days follow-up)
Milligan	2022	CardioMEMS	Cohort study	…	NYHA III HF and an implanted CardioMEMS	53	Patient own historic control	IRR 0.48; 95% CI 0.32–0.70; *p* < 0.001 (6 months post-implant versus 6 months pre-implant) IRR 0.56; 95% CI 0.41–0.76; *p* < 0.001 (12 months post-implant versus 12 months pre-implant)
Mullens	2020	Cordella	Non-randomized trial	SIRONA	NYHA III HF and HFH < 12 months	15	…	…
Sharif	2022	Cordella	Non-randomized trial	SIRONA 2	NYHA III HF and HFH < 12 months or elevated NT-proBNP/BNP	75	…	8 HFH (90 days follow-up) 11 HFH (6 months follow-up)

HF: Heart Failure; HFH: Heart Failure-Related Hospitalization; NYHA: New York Heart Association; CardioMEMS: CardioMEMS HF system; Cordella: Cordella HF system; HR: Hazard Ratio; IRR: Incidence Rate Ratio; 95% CI: 95% Confidence Interval; RCT: Randomized Controlled Trial.

## Data Availability

Not applicable.
